# “Am I carrier?” The patient's lived experience of thrombophilia genetic screening and its outcome

**DOI:** 10.1080/21642850.2014.918512

**Published:** 2014-06-04

**Authors:** Guendalina Graffigna, Daniela Leone, Elena Vegni

**Affiliations:** ^a^ Faculty of Psychology, Università Cattolica del Sacro Cuore, Milan, Italy; ^b^ Department of Health Science, Università degli Studi di Milano, Milan, Italy

**Keywords:** genetics, testing outcomes, hereditary diseases, interpretative phenomenological analysis (IPA), thrombophilia, illness perception

## Abstract

How do patients with thrombophilia experience a physician's request to undergo a genetic test? How do they experience the test outcome? To answer these questions, we conducted an interpretative phenomenological analysis study, based on 10 in-depth interviews with patients who underwent genetic testing for thrombophilia in Italy, half with positive and half with negative results. The experience of undergoing genetic screening for thrombophilia plays an important role in reconfiguring patients' signification of their illness experience. A positive outcome becomes a cue to reorganize in a more adaptive way the illness meaning at the cognitive and emotive levels, whereas a negative outcome appears more distressing and confusing. As a clinical implication of the study, clinicians should consider communicating carefully with the patients regardless from the positive/negative test results and they should explore the patient's specific reaction and understanding of test result.

## 1. Introduction

Understanding patients' perspectives and lived experiences is undoubtedly an asset in improving efficiency in health interventions and promoting clinical adherence (Zolnierek & DiMatteo, 2009). The concept of illness perceptions as delineated by Leventhal, Nerenz, and Steele (1984) has been widely used to describe the organized representations or beliefs that patients have about their illness. The experience of a symptom is the starting signal of a cognitive search to understand the health threat. This process results in the individual developing a perception of the illness that comprises a number of distinct but interrelated themes: identity (the illness label and number of symptoms), cause, timeline, consequences, perceived control, and emotional representations (the emotional reactions to the experience of a medical problem). These perceptions have been found to be important determinants of behavior and have been associated with a number of important outcomes, such as treatment adherence and functional recovery (Machin, 2003; Mannucci, 2001). In particular, the diagnosis of any chronic medical problem confronts individuals with a collection of tasks necessary for both physical and psychological adjustments. These can involve acceptance of a certain amount of loss of function and might require the acquisition of new skills and changes to daily routines for the patient to manage the illness symptoms or cope with the demands of treatment (Hertzberg, 2005). Furthermore genetic causes of an illness status elicit complex patient processes of meaning making that need to be explored in order to understand their consequences on patients’ behaviors and attitudes to health conducts (Senior, Smith, Michie, & Marteau, 2002).

Eliciting the patient's view of his or her illness during the clinical encounter offers the potential for a better understanding of (mal)adaptive response to illness and treatment and has implications for medical practice. Several studies provide support for the relationship between the illness representation and a range of outcomes such as functional adaptation, quality of life, and treatment compliance for individuals with chronic illnesses such as cardiac disorders (Constans, Boulon, Solanilla, & Conri, 2008), type 2 diabetes (Hellmann, Leslie, & Moll, 2003), rheumatoid arthritis (Gartner, Weber, & Eichinger, 2008), and chronic-degenerative illnesses such as cancer (Bank, Scavenius, Buller, & Middeldorp, 2004; Eichinger, 2009; Heshka, Palleschi, Howley, Wilson, & Wells, 2008).

Researchers have also discussed patients' perceptions of screening tests, evincing the importance of the cognitive and the emotional experiences of patients in their comprehension and use of test results. This is particularly important when genetic screenings are concerned: in other words it is important to understand how patients give sense to their genetic illness condition and how this impacts on their coping strategies (Riggs & Giuliano, 2007). Enrollment in health screening might constitute a risk of distress in healthy adults (Howard, Balneaves, Bottorff, & Rodney, 2011; Stoate, 1989). The general effect of the screening in terms of reduction of anxiety and concern seems to depend not on test results but on the baseline assessment of perceived anxiety and/or concern before the test (Swanson, McIntosh, Power, & Dobson, 1996). For instance, of 127 people receiving negative results following predictive genetic testing for familial adenomotous polyposis (FAP), the more anxious people were about the chance of developing FAP, the more likely they were to perceive the test results as uncertain and threatening (Michie et al., 2002). In a recent study involving people without a high cardiovascular risk score, participants in good health perceived negative test results as a way to eliminate worries and confirm their lifestyle (Nielsen, Dyhr, Lauritzen, & Malterud, 2009). Similar evidences, but with a very different perception by patients, were achieved in a study exploring the effect of genetic testing for familial hypercholesterolemia which demonstrated that patients receiving a negative result may “feel in limbo” (Hilgart, Mercer, & Thirlaway, 2013).

Although studied less frequently, the individual's perception (ideas, concerns, and feelings) of a screening before and after experiencing it might deeply influence its usefulness and clinical impact. Studying patients' lived experiences and meaning-making processes related to health screening thus deserves increased attention because these emotive and cognitive processes can play an important role not only in influencing patients' attitudes toward the diagnosis (Mendes, Santos, & Sousa, 2011; Tluczek, Orland, & Cavanagh, 2011) but also in enhancing or hindering their willingness to undergo a test, with important clinical consequences. This is particularly evident in the case of preventive medicine. Several studies have underlined that rates of preventive screenings are still below the recommended levels; for example, cancer (Katalinic & Plestina, 2010), papillomavirus (Hill & Gick, 2011), and even HIV/AIDS (Graffigna & Olson, 2009). The problem of knowing one's own health status, and thus the emotional frame in which a health screening and its outcome are lived and perceived, is an important factor that influences people's attitude toward care and cure, and needs to be taken further into account.

### 1.1. Thrombophilia testing

Exploring how patients perceive thrombophilia screening appears interesting because of the characteristics of this disease as the following will explain. Thrombophilia (or hypercoagulability) is the propensity to develop potentially dangerous thromboses (blood clots) because of a hereditary abnormality in the coagulation system. Additional risk factors such as obesity or diabetes can enhance the chances of developing the disease. The discovery of genetic risk factors for venous thrombosis, such as factor V Leiden and the prothrombin gene mutation 20210A, led to a widespread clinical application of genetic screening that included healthy individuals with no personal or family history of venous thromboembolism, such as the screening of healthy women before the prescription of oral contraceptives (Mannucci, 2001; Martinelli, 2003). However, the clinical utility of thrombophilia testing (for a deeper understanding of specificity and sensitivity of the test see Tripodi, 2005) is still a matter of debate because of the unclear clinical and economic implications (Baglin, 2003). Although the test has the ability to reveal whether one individual is more likely than another to suffer a thrombosis, data on its ability to predict a recurrence in patients who have already had thromboses are conflicting (Machin, 2003; Martinelli, 2003). Opponents of widespread thrombophilia screening also argue that testing all patients generates needless anxiety, causing negative effects on psychological status and social stigmatization (Eichinger, 2009; Gartner et al., 2008; Hellmann et al., 2003; Machin, 2003; Mannucci, 2001).

Genetic testing does not necessarily cause psychological anxiety, but neither does it necessarily motivate behavioral change (Heshka, Palleschi, Howley, et al., 2008; Heshka, Palleschi, Wilson, et al., 2008). However, some researchers assessing the psychological impact of awareness of being a thrombophilia carrier (Lindqvist & Dahalback, 2003; Van Korlaar et al., 2005) found that participants reported a belief in the psychological and health benefits of testing and of being informed of their status. These pieces of evidence underscore the complexities of the importance of the test for thrombophilia and the contradictory value it can have in different conditions; that is, having a thrombotic event in one's clinical history or being at risk of having thrombophilia because of a genetic mutation. As a consequence, further studies aimed at deepening subjective experiences of being tested for thrombophilia (and of receiving the test outcomes) are needed to support effective clinical practices and preventive strategies. Only a deep understanding of the subjective meanings that patients attribute not only to their clinical situation but also to the fact of undergoing a genetic test to understand the nature of their disease can orient more ecological and efficient medical practices and communication strategies. Furthermore, studies conducted so far on the experience of thrombophilia screening have focused only on the lived experience of patients who received a positive outcome to the genetic test (Moore, Norman, Harris, & Makris, 2008), whereas previous studies on other issues have shown the importance of considering the psychological and behavioral effects of a negative result; for example, in cardiovascular risk (Nielsen et al., 2009) or the genetic test for familial adenomatous polyposis (Michie et al., 2002). In a previous qualitative study by our group (Vegni, Leone, Graffigna, Faioni, & Moja, 2013) about the way patients perceive the thrombophilia test, results suggested that a complex view of the test may be the result of an intricate and perhaps peculiar conception of an “illness of the blood”: not all the interviewees had a thrombotic event, but they all perceived a problem in the blood, materialized by the test. The thrombophilia test itself is perceived as a tool for knowledge, and in particular knowing for the sake of doing, with the risk of frustration. The study could not distinguish, however, how far those results may depend on positive vs. negative results, or if the quality of the results influences the experience of a peculiar view of the disease and/or the test.

## 2. The study

### 2.1. Method and research design

The study was designed according to interpretative phenomenological analysis (IPA) (Eatough & Smith, 2008), which among the different qualitative methods, appears the best heuristic approach for casting light on the constellation of meanings and emotions that frame the lived experience of being tested for thrombophilia and of receiving the test outcomes (Chapman & Smith, 2002). This method involves the detailed analysis of individuals' lived experiences and of the way in which they attribute sense to those experiences and it allows the researcher to grasp the essence of how people experience and understand the world (Conroy, 2003; Goodman, Morrissey, Graham, & Bossingham, 2005; Richards & Morse, 2007). Furthermore, through the analytical process of IPA it is possible to uncover implicit beliefs and emotions that are carried by interview transcripts, although patients are not completely cognitively aware of those psychological states (Smith, Jarman, & Osborn, 1999).

### 2.2. Study objectives

Our qualitative study was aimed at (a) illuminating the meanings (even the most latent and implicit) that patients attribute to their experience of genetic screening for thrombophilia, with particular attention to worries and doubts related to it; and, in particular, (b) exploring in depth how patients experience the outcome of genetic tests in the case of a positive versus a negative result. Findings from this study can contribute to improvements in clinical practice and healthcare providers' communication strategies in the case of thrombophilia screening.

### 2.3. Participants

This study is part of a larger project exploring the perception of the illness experience of thrombophilia that enrolled 26 patients with thrombophilia (Vegni et al., 2013). The inclusion criterion for the overall project was that the patient had been screened for thrombophilia at the hematology unit of San Paolo Hospital of Milan between 1 year and 6 months previously. Out of this broader sample, we re-interviewed 10 patients to more fully understand the meanings they attributed to the genetic test in general and, in particular, to its outcomes. Considering the objectives of the study, we opted both for patients who received a positive outcome in the genetic test and for those who received a negative result. Out of the broader sample, only four patients who received a positive outcome and six patients who received a negative answer appeared eligible for this second phase of the study and consented to undergo a second interview. In order to increase clinical homogeneity we included only patients who experienced a thrombotic episode as a reason for the testing. Those with positive outcomes had a mean age of 69 years (range 59–76 years). The mean age of patients with negative outcomes was 53 years (range 44–66 years). All recruited patients were female (Table 1).

**Table 1.** Participants' socio-demographic and clinical data.

Participant	Test result	Sex	Age	Profession
P1	Positive	F	69	Retired
P2	Negative	F	52	Domestic servant
P3	Positive	F	59	Housewife
P4	Negative	F	66	Retired
P5	Negative	F	65	Retired
P6	Positive	F	70	Retired
P7	Positive	F	76	Seamstress
P8	Negative	F	55	Housewife
P9	Negative	F	44	Health worker
P10	Negative	F	37	Housewife

The selection of these patients was based on their ability to reflect on their experience and to articulate accounts of it during the first interview: On the whole 10 patients were re-interviewed and their narrative appeared sufficient to cover our objectives of study. The second interviews took place in a maximum time frame of four weeks after the first interviews, so that the experience of genetic testing and receiving its outcome was still relatively recent and relevant in patients' experience. Results that follow in this paper are partially overlapped with the general findings regarding the experience of being screened for thrombophilia. During the first interview, suggestions regarding the meaning of positive/negative test results were briefly discussed, and the findings are reported elsewhere (Vegni et al., 2013). The purpose of the second interviews was to more deeply understand the emotional and representational reactions of patients to the genetic test outcomes, by casting light on the complex systems of meanings that orient patients' attitudes toward their illness.

### 2.4. Ethical concerns

The study was approved by the ethics committee of the San Paolo University Hospital. Before being included in the study, patients were informed about the research aims and procedure by a professional hematologist of the San Paolo University hematological unit together with a researcher responsible for the study. Patients enrolled by signing a detailed consent form, and they were free to leave the study if they felt uncomfortable during the interview. During transcription, references to names and/or places were removed and anonymized data were analyzed to respect participants' data privacy

### 2.5. Data collection

Data collection for our study was based on in-depth semistructured interviews, which constitute the most widely selected data collection strategy for the IPA approach (Eatough & Smith, 2008). These second interviews were conducted by the authors (DL and EV) at the Department of Clinical Psychology of San Paolo Hospital of Milan and lasted about one hour. Patients were asked to describe the experience of being screened for thrombophilia and the experience of receiving information about the test outcome. The interviews were semistructured to allow participants to expand on other areas they deemed important (see in Appendix some example of the questions included in the interview guide). Data collection in IPA is dialogic (Eatough & Smith, 2008) because the participant has an important role in determining the course of the interview. The interviews were recorded and transcribed.

### 2.6. Data analysis

We analyzed the whole verbatim transcripts of the selected participants' interviews (both the first and the second ones) according to IPA procedure (Smith, Flowers, & Larkin, 2009). First interviews were included for completeness but analyzed with a different focus (i.e. on the meaning-making process related to the test outcomes) and procedure. Length of the transcribed and analyzed interview was in a range between 25′25′′ and 48′ 23′′ (i.e. second interviews were generally a bit longer than the firsts; the mean length of the interviews analyzed was of 34′22″). Analysis in IPA is an iterative, inductive process that starts from the detailed reading of the transcript aimed at gaining a holistic understanding of all collected narratives. In this early phase of the analysis the researcher takes notes (memos) of his or her understanding of the text and preliminary insights about the meanings that participants attribute to their experience (Richards & Morse, 2007). The analysis then proceeds with several rereadings of the interview transcripts and a more detailed, step-by-step coding of the texts to detect and to describe the main analytic themes present in the texts and their interconnections. The researcher continues the analysis while writing the study's main results, producing, finally, a reconstruction of participants' lived experience of the phenomenon and of the meanings they attributed to it.

In our study three researchers (GG, DL, and EV – the same research team involved in the broader study) conducted the analysis contemporaneously through parallel blind coding of all the transcripts. We adopted an independent co-coding approach to ensure rigor of the analysis process (Morse, Barrett, Mayan, Olson, & Spiers, 2002). Preliminary results of the analysis were discussed in depth during several research meetings to gain agreement. This process allowed a better research reflexivity during the analysis process, thanks to the sharing of emerging interpretations among the colleagues involved in the analysis; this also sustained a better re-elaboration and abstraction from the data in the final phases of the analysis. In addition to these debriefing meetings among the three researchers, at a final stage we discussed the findings with a hematologist in order to give them a more clear clinical and practical meaning. Finally, all themes (i.e. one super-ordinated theme, then articulated into three sub-themes, as represented in Table 2 and described in the following paragraphs) emerged from the analysis will be described in the following paragraphs and substantiated by reporting significant and exemplificative excerpts from interviews, as required in the IPA analytical procedure (Smith, 2011)

**Table 2.** Thematic structure of results.

Main theme	Sub-themes	Exemplificative verbatims
The reconfiguration of patients' signification of their illness experience	The test as a cognitive organizer of the illness representation	*It is like a Calvary. A long, never ending list of exams * … * counting then that I also have other health problems!* (P7)
		*This disease is an endless torment, since it is because of my genes, I will never disappear* (P6)
		*I'm very religious and some time I think that it was God, this was like a divine punishment”* (P7)
		*I found I am carrier. I always have been predisposed to thrombophilia.* (P6)
		*I have a genetic disease. I was probably born with it.* (P1)
		*Honestly I would have preferred to be genetically predisposed to the disease, because at least you put a full stop on this continuous peregrination of exams and clinical encounters to know what are you suffering from* (P9)
		*If I had knew to be heridatarian I would have been distressed on a had, but on the other hand it would have been a relief … because it gives a name to your problem* (P8)
	The test as a cognitive organizer of the illness representation	*I waited more than a month to know the test result. It has been a very long period. I was anxious to know the result because this idea of genetics is quite scaring* (P6)
		*A long time without knowing anything. * … * I was very worried because I thought that they took so much time was because my situation was more serious than I expected.* (P3)*Waiting for the result was distressing, as it is for all the exams. But I was also exhausted because I didn't know what were the causes of my problem. I had a sort of mixed feelings: fear but also hurry to know it, to receive a final answer* (P1)
		*It is not said that everybody is alike, but if in your family someone had thrombophilia, it is possible that someone else will have the problem: The blood is the same* (P1)
		*My mum had the same problem, I saw her and her story, in some ways I can tell what will happen to me* (P6)
		*They told me that also my children could have been at risk, so they asked also them to do the exam … I think that is a sort of prevention* (P3)
		*Now I worry that my daughter may experience the same episode … I want her to do my same exam to prevent it* (P6)
		*I knew it, it is impossible to understand what I have* (P5)
		*I must resign myself: I have an untreatable disease* (P8)
		*Not knowing what you are suffering for is very distressing, even more that knowing what it is. I'm very tired by this situation* (P10)
		*After the test I haven't done anymore exploration: I feel better, plus now I know that my disease is not genetic* (P8)
		*I feel more relaxed now. I think that the only right attitude is to be more lighthearted * … * it is not worthwhile to worry too much* (P2)
	The pragmatic value of the test outcome	*The point is that I don't know what to do now * … * I'm carrier * … * and so what?* (P1)
		*At the beginning I was relieved at knowing at least the origin of my illness * … * but then you discover that is a terrible illness for which you cannot do nothing * … * so maybe it is better to not know it!* (P3)
		*Now that I know that I'm not hereditarian I honestly don't know if it is a good or a bad news. In the practices I don't know what to do* (P4)
		*It is one of the several exams, with one of the several outcomes that you don't really understand in the practice. I hope they will let me know what I should do* (P5)
		*Honestly it is not very clear to me the usefulness of this exam* (P2)
		*The doctor communicated the outcome to me, but he didn't explain anything else. Now I don't know what I shall do* (P9)
		*Maybe it is better not to know * … * because after you know it you cannot do anything. I think this is like an anticipatory information about possible relapses* (P7)
		*I want to know: Truth is hard * … * but it is better to know it. However I still have doubts: this is an answer about the causes of the problem, but what about its possible evolution?* (P2)

## 3. Results

The analysis conducted allowed to retrieve a principal theme, common to all interviews: “the reconfiguration of patients’ signification of their illness experience”. This theme appeared crucial and pivotal in interviewees' stories, and fundamental to understand the impact of the thrombophilia genetic test outcome on patients' attitudes and representation about the disease. This theme appears to saturate principal insights pertaining to the analyzed interviews. However this theme is conceivable as a super-ordinate theme, internally articulable in the following three sub-themes (i.e. “the test as a cognitive organizer of the illness representation*”, “*the test as an emotional organizer*”,* and *“*the pragmatic value of the test outcome*”*) that specifically describe this reconfiguration process at the cognitive, emotive, and behavioral levels (Table 2). In the following paragraphs we shall describe in detail these themes by reporting some exemplificative interviewees' quotations in order to better illuminate our interpretations.

### 3.1. The reconfiguration of patients’ signification of their illness experience

According to our interviewees' narratives, the experience of the genetic test for all interviewees is inserted in a long history of clinical examinations, suffering, and continuous peregrinations from one hospital to another in an attempt to discover the causes of their illness.

I've already had 14 surgeries, you can understand! (P4)

My story has started since I was young: I have undergone so many exams in my life. (P1)

Because of a shared common confusion related to the illness experience and to the difficulty of giving meaning to it, the genetic test assumes a particular representational and emotive value for patients undergoing it. In both cases, however, the test seems to play the role of a potential “meaning organizer”: patients, particularly those who tested positive, experience the test as, if not the answer to their doubts (and to their hematologist's doubts), at least as an important milestone in their continuous search for an answer regarding the nature of their disease. In other words, the test outcome offers to the patients an important cue to better understand and attribute meaning to their illness and to the reasons of their sufferance.

Knowing that I'm heridatarian was a bit distressing at the beginning, but at least now I know why I'm suffering of it. I was born with it, that's the problem.! (P1)

At least I know that it is not genetic, so probably I can treat it (P10)

The test outcomes seem to perform the function of organizing meaning at several levels (cognitive, emotional, and pragmatic) by reframing patients' illness representations, as described in the following paragraphs. We shall argue on these in the following paragraphs.

### 3.2. The test as a cognitive organizer of the illness representation

The genetic test becomes the occasion to circumscribe and piece together the different clues of one's illness problem to understand its nature. The genetic test assumes a catalytic function that patients who had positive outcomes ascribed different meanings compared to those with negative ones, as we shall argue in the following paragraphs.

For patients who tested positive, the discovery of being a carrier changed the perspective from which they looked at their illness. In their narratives, the experience of the test becomes the focus of their story. The genetic nature of thrombophilia is read as the origin not only of their illness but also of their suffering (a feeling that they define as a “Calvary” (P7); an “agony” (P3); a “endless torment”(P6); a “divine punishment” (P7) to underline the intense discomfort linked to the disease and to the continuous examinations experienced in the attempt to find their disease origin; representation that also shows a general passivity of the patient and a general acceptations of their condition).

It is like a Calvary. A long, never ending list of exams … counting then that I also have other health problems! (P7)

This disease is an endless torment, since it is because of my genes, it will never disappear (P6)

I'm very religious and some time I think that it was God, this was like a divine punishment” (P7)

Thus, at the cognitive level the test outcome becomes the turning point of patients' experience, the pivot around which their illness story is reconstructed and re-understood.

I found I am carrier. I always have been predisposed to thrombophilia. (P6)

I have a genetic disease. I was probably born with it. (P1)

For patients who received a test negative outcome, this representational function is less prominent and patients appear caught in a cognitive-emotional conflict: cognitions and emotions in relation to the testing process result are inextricably linked, particularly among the patients who – when receiving a negative outcome – felt frustration in regard to their knowledge expectation. These patients expressed their strong investment in the test, expecting it to be a key to understanding their illness state and putting a “full stop” to the “darkness of the disease experience” (P10). But the negative outcome is lived as another “lost occasion” (P3) and as a further confirmation of their indeterminate state.

Honestly I would have preferred to be genetically predisposed to the disease, because at least you put a full stop on this continuous peregrination of exams and clinical encounters to know what are you suffering from (P9)

If I had knew to be heridatarian I would have been distressed on the one hand, but on the other hand it would have been a relief … because it gives a name to your problem (P8)

The negative outcome of the genetic test, thus, is experienced by patients as a last occasion to gain a final cognitive cue on their condition. In this regard, some participants referred to cancer as a more critical condition at the physiopathologic level but preferable at the cognitive and representational levels (because at least it is perceived as well as known, recognizable, and treatable).

At least if you have cancer you know what are you suffer from! (P2)

Cancer is a treatable disease because doctors know its cause and origin. In my case it is not possible to find the reasons why I have all these problems. (P10)

### 3.3. The test as an emotional organizer

The representational function played by the genetic test is strongly linked to a change at the emotive level: all participants expressed specific feelings related to the test outcome and to the ensuing representational reconfiguration of their disease. However the emotional connotation of the disease experience appears distinctive and different between participants who received positive and negative outcomes.

Patients with positive results reported the experience of great anxiety mixed with an ambivalent feeling resulting from worry about being certain of having a genetic disease (perceived thus as definitive, “unchangeable”, P7) and also the hope of finding a cue to disentangle a confused pathological situation.

I waited more than a month to know the test result. It has been a very long period. I was anxious to know the result because this idea of genetics is quite scaring (P6)

A long time without knowing anything. … I was very worried because I thought that they took so much time was because my situation was more serious than I expected. (P3.)Waiting for the result was distressing, as it is for all the exams. But I was also exhausted because I didn't know what were the causes of my problem. I had a sort of mixed feelings: fear but also hurry to know it, to receive a final answer (P1)

The news of the test outcome is lived by all “positive” interviewees as the source of a renewed sense of control over the disease and its evolution, at least at the emotive and representational levels (as discussed in the following paragraph). From this perspective, the positive outcome of the genetic test is perceived in terms of a “family trait”, (P7) and the disease assumes a more neutral representation (i.e. less worrying). The genetic test is emotively perceived as a test for tracking the transmission of the disease from one generation to another. This helps the person affected in rendering the problem concrete and in perceiving a renewed power over it.

It is not said that everybody is alike, but if in your family someone had thrombophilia, it is possible that someone else will have the problem: The blood is the same (P1)

My mum had the same problem, I saw her and her story, in some ways I can tell what will happen to me (P6)

For these patients, thus, taking care of thrombophilia includes taking care of the next generation. They reflect on how to protect their children in the future. It follows that the test is perceived as a “preventive” (P1) instrument for detecting risk factors and is lived with less anxiety.

They told me that also my children could have been at risk, so they asked also them to do the exam … I think that is a sort of prevention (P3)

Now I worry that my daughter may experience the same episode … I want her to do my same exam to prevent it (P6)

A different emotive reaction is shared by patients who tested negative. As mentioned previously, this was perceived as a further failure in understanding the reasons for their suffering, and it is received with a sense of disillusion and hopelessness. The test is lived by these patients as a “defeat” and as the confirmation of the severity and inexorability of thrombophilia.

I knew it, it is impossible to understand what I have (P5)

I must resign myself: I have an untreatable disease (P8)

Not knowing what you are suffering for is very distressing, even more that knowing what it is. I'm very tired by this situation (P10)

This follows an enhanced tendency to delegate to the healthcare system the management of their state (i.e. decisions about treatment and diagnostic assessment). Furthermore, for some patients with negative screening results, this attitude was also attributable to the sense of relief they experienced after the test outcome: These participants interpreted their “not being a carrier” as “not being at risk”, lessening their motivation to the research into their disease's causes.

After the test I haven't done anymore exploration: I feel better, plus now I know that my disease is not genetic (P8)

I feel more relaxed now. I think that the only right attitude is to be more lighthearted … it is not worthwhile to worry too much (P2)

### 3.4. The pragmatic value of the test outcome

As a possible consequence of these representational and emotive functions (particularly evident in the case of patients who tested positive), it seems that at the pragmatic level there is or remains nothing to do: knowing themselves to be “carriers” of thrombophilia does not seem to affect patients' daily practices. Both groups of participants (the “positive” and the “negative” at the test) show the same inability to “use” the diagnosis received from the test to orient their practices. Knowing the nature of the disease, indeed, is not enough for the patient to develop strategies that can help in managing the disease evolution.

The point is that I don't know what to do now … I'm carrier … and so what? (P1)

At the beginning I was relieved at knowing at least the origin of my illness … but then you discover that is a terrible illness for which you cannot do nothing … so maybe it is better to not know it! (P3)

Now that I know that I'm not hereditarian I honestly don't know if it is a good or a bad news. In the practices I don't know what to do (P4)

It is one of the several exams, with one of the several outcomes that you don't really understand in the practice. I hope they will let me know what I should do (P5)

In fact, although at the emotive level patients with positive screening results declared a renewed sense of control over their disease and reported a new determination to keep an active role in their care and cure management, in practice they did not differ from participants with negative tests. All participants seem to find difficulties in grasping the pragmatic utility of the genetic diagnosis for thrombophilia. In this frame, the patient finds it difficult to decode the practical value of the genetic test outcomes.

Honestly it is not very clear to me the usefulness of this exam (P2)

The doctor communicated the outcome to me, but he didn't explain anything else. Now I don't know what I shall do (P9)

In fact, it is unclear whether the test is aimed to cast light on the causes of the thrombotic event or if it is aimed at identifying patients' risk of relapse.

Maybe it is better not to know … because after you know it you cannot do anything. I think this is like an anticipatory information about possible relapses (P7)

I want to know: Truth is hard … but it is better to know it. However I still have doubts: this is an answer about the causes of the problem, but what about its possible evolution? (P2)

## 4. Discussion

The evidence that emerged from this study underlined that the test assumes important emotive and symbolic meanings in the patients' lived experience. In fact, rather than the test results (and previous to them), the experience of undergoing genetic screening for thrombophilia was charged with important patients' expectations and played a central role in reconfiguring signification processes related to their illness experience. Several studies have been conducted in other disease domains in order to assess the original differences between testing and non-testing individuals according to motivation and beliefs related to their health status (see for instance: Evers-Kiebooms, Welkenhuysen, Claes, Decruyenaere, & Denayer, 2000; Taylor, 2005), while there is a lack of this kind of studies in the field of genetic assessment of thrombophilia, both because the testing practice is still at its infancy and controversial. In this frame, our study, although exploratory and based on a small sample, seems to underline that the different meanings attributed to the testing experience itself seem to be independent of individual characteristics of the patients.

This insight appears interesting, not only because it suggests shifting the debate from the psychological consequences of receiving a screening outcome (Broadstock, Michie, & Marteau, 2000; Levine, 1991; McCann et al., 2009; Michie et al., 2002; Nielsen, Dyhr, Lauritzen, & Malterud, 2004, 2009; Stoate, 1989) to the meanings attributed to the screening experience itself, but also because it suggests interesting clinical implications that need to be considered to improve health provider handling of requests to patients for thrombophilia testing. In this regard it would be interesting to verify whether a similar experience can be detected in relation to genetic screening for other diseases or if this is typical only of the genetic test for thrombophilia.

Within the specific scope of the genetic test for thrombophilia, this study showed that positive and negative outcomes have different impacts on patients' lived experience of the disease. In particular, receiving a positive outcome, rather than being a source of anxiety and distress (Bank et al., 2004; Eichinger, 2009; Hellmann et al., 2003; Machin, 2003; Mannucci, 2001), becomes for patients a new cue to reorganize the illness meanings at both the representational and the emotive levels. A negative outcome, however, has a disorienting effect on patients' experiences. It is understood as one of many failed attempts to understand the nature of their disease and, with a cognitive jump, as a confirmation of its inexorability and intractability. This issue is partially contradicted by the observation that, regardless of the positive or negative result, there is a sense of helplessness and disempowerment experienced by patients, and the practical value of the genetic testing is questionable to participants, regardless of the outcome. Looking at previous studies on genetic test perception in different clinical contexts this at least ambiguous utility perceived by patient may be detected.

For instance, in a very different clinical context, a study conducted on the effects of genetic testing on patients' beliefs and motivation in heart disease (Senior et al., 2002) underlined that a positive test outcome tends to have a “paralyzing” psychological effect for patients, since it would foster the perception of illness inexorability and of self-inefficacy (Riggs & Giuliano, 2007). In a study exploring the effect of genetic testing for familial hypercholesterolemia, patients receiving a negative result may “feel in limbo” (Hilgart et al., 2013), suggesting, as in our study, that a negative result may not be reassuring for the patient. As a consequence of the clinical implication of these findings, clinicians should consider to communicate carefully with the patients regardless of the positive/negative test results and they should be aware that a negative result could not be perceived as a “good” result for the patient. Understanding the specific reaction of the patient while he/she is listening to the test result is important in order to assure the effectiveness of the conversation.

Anxiety related to the genetic screening outcomes (focused in the ongoing debate; Almqvist, Brinkmann, Wiggins, & Hayden, 2003; Lodder et al., 2001; Shaw, Abrams, & Marteau, 1999; Swanson et al., 1996) needs further clarifications. Our study showed that it is an emotion common to all interviewees, not only those who received positive results. However, anxiety is associated with different meanings in the two groups of patients. For patients who received a positive result, the test outcome works as an anxiety reliever because it constitutes a first answer in their long search for the meaning and cause of thrombophilia. In patients who received a negative outcome, however, this search for the causes of thrombophilia remains open and this serves to maintain a high level of anxiety in patients. As a consequence, rather than focusing on the level of anxiety related to the genetic test outcomes, researchers might explore strategies that physicians can adopt to manage patients' anxiety. This also suggests the importance of further reflection on the most suitable strategies to manage doctor–patient communication related to the request to undergo a genetic test for thrombophilia, in relation to both the way the doctor should propose the test to the patient and the information that he or she should provide in the informed consent (What information does the patient need to be fully aware of the genetic screenings implications? What information is crucial to make the patient able to make a decision in this regard?).

Finally, the effects of patients' decoding of the genetic nature of thrombophilia (positive test outcome) in terms of a “family trait” appear coherent with previous studies conducted in other disease domains. These studies have underlined how the symbolic equation between “genetics” and “familiarity” tends to diminish patients' fatalism and sense of self-inefficacy toward genetic illness conditions (Riggs & Giuliano, 2007).

Physician communication regarding the genetic test for thrombophilia and its outcome is framed in a multifaceted symbolic and emotional world that needs to be taken into account. As a consequence, implicit and cursory medical communication might generate dangerous misunderstandings on the part of patients. Poor medical communication might produce messages that are difficult for the patients to decode and become the object of wide emotional resonance. For example, Marteau and Croyle (1998) showed that adverse psychological reactions were uncommon when testing was accompanied, before and after, by clear information and emotional support. A tailored process of patient education, according to a patient-centered approach (Stewart et al., 1995), which appears still unexplored in the thrombophilia studies, could reduce the risks of misunderstanding the meaning of the test. In this regard, it would be interesting to study doctor–patient conversational exchanges in real medical settings to explore the conversational strategies used by the doctor when requesting that the patient undergo the genetic test (i.e. is the test imposed or proposed?) and to explore patients' reactions and negotiation strategies. This exploration in the context of medical encounters could cast further light on patients' understanding of genetic testing and its outcomes and could offer important insights to orient physician communication strategies.

Finally, from a methodological perspective, evidence from this study underscores the value of an attentive and deep analysis of the patient's lived experience of thrombophilia screening and of the psychological nuances that characterize this experience. Following from this is the usefulness of research approaches such as qualitative methods that are flexible and thus enable the researcher to grasp patients' symbolic and experiential world. From this perspective, IPA is confirmed as the research approach best suited to illuminating the most fluid and elusive features of the illness and care experience. Although many studies have been conducted on thrombophilia (Bank et al., 2004; Moore et al., 2008; Saukko, Ellard, Richards, Shepherd, & Campbell, 2007; Saukko, Richards, Shepherd, & Campbell, 2006), the use of IPA allowed us to discover a new perspective on the phenomenon and on how patients live it. In fact, it allowed us to go deeper in the analysis of this illness experience and to discover the complex weaving of symbolic meanings and representations that patients attribute to it, a discovery that would have been impossible with other research approaches, such as quantitative ones, which permit researchers to measure and generalize manifestation of a phenomenon but not to catch its essence and its complex implicit structure.

## 5. Conclusions and study limits

This study, which is part of a more complex project aimed at exploring patients' experience of thrombophilia (Vegni et al., 2013), offers some preliminary insights into patients' symbolic and emotional framing of thrombophilia genetic screening and its outcomes. Furthermore, the study can be considered as an interesting testing ground for confirming the heuristic value of IPA to further patients' lived experiences at the referential and at the implicit levels. However, although relevant, the evidence that emerged in this study is preliminary and needs further investigation. In particular, it would be interesting to continue this exploration by interviewing a wider and more varied sample of patients to catch potential articulation and typologies of the lived experiences of the genetic test, different experiences that might be linked to a varied combination of the symbolic-experiential words disclosed by this preliminary research.

Our sample was quite homogeneous regarding sociocultural characteristics and previous illness history and in particular, the accepting participants were only females. It would thus be worthwhile to explore more fully how the experience of undergoing a genetic test for thrombophilia is lived by male patients and subjects who have different backgrounds and different concomitant health problems. It would also be important to explore the influence that the length of time a patient has been undergoing diagnostics has on the meaning-making process about the genetic test for thrombophilia by following patients over time in a longitudinal study. Furthermore, to better support clinical practice and communication strategies regarding thrombophilia screening, it would be worthwhile to study patients' experiences through conversational analysis of the exchanges that concretely take place between the doctor and the patient when thrombophilia screening (or its outcome) is communicated. Finally, it would be valuable to compare findings achieved in this study with those from other qualitative, IPA studies aimed at exploring patients' lived experience of genetic testing related to other diseases to explore whether some meaning-making processes illuminated in this study are constant.

## Appendix. Interview schedule scheme

First interview:

- started with a widely open question: “What about your background in the hematology Unit?”.
- Followed by more specific questions about the genetic test: “What about the test you did/mentioned for a genetic testing for thrombophilia?”/ “What the doctors explained to you about the test?”/ “What did you understand about the test”?/“How did you feel doing the test”?/ “Was the communication with your doctor satisfying for you”?

In the case of a second interview we asked participants to discuss the results regarding in particular the genetic test and the meaning they attributed to it in the light of positive vs negative results: “How did you know about the test result?”/“What did you understand about the result?”/“How did you feel resulting positive/negative?”
